# Increased plasma D-dimer levels may be a promising indicator for diabetic peripheral neuropathy in type 2 diabetes

**DOI:** 10.3389/fendo.2022.930271

**Published:** 2022-08-23

**Authors:** Lei Zhuang, Chao Yu, Feng Xu, Li-hua Zhao, Xiao-hua Wang, Chun-hua Wang, Li-yan Ning, Xiu-lin Zhang, Dong-mei Zhang, Xue-qin Wang, Jian-bin Su

**Affiliations:** ^1^ Department of Endocrinology, Second People’s Hospital of Nantong City, Nantong, China; ^2^ Department of Clinical Laboratory, Affiliated Hospital 2 of Nantong University, and First People’s Hospital of Nantong City, Nantong, China; ^3^ Department of Endocrinology, Affiliated Hospital 2 of Nantong University, and First People’s Hospital of Nantong City, Nantong, China; ^4^ Department of Administration, Affiliated Hospital 2 of Nantong University, and First People’s Hospital of Nantong City, Nantong, China; ^5^ Medical Research Center, Affiliated Hospital 2 of Nantong University, and First People’s Hospital of Nantong City, Nantong, China

**Keywords:** D-dimer, neuropathy, type 2 diabetes, risk, diagnosis

## Abstract

**Background:**

Increased plasma D-dimer levels have been reported to be associated with a range of adverse health outcomes. This study aimed to determine whether plasma D-dimer is connected to diabetic peripheral neuropathy (DPN) in patients with type 2 diabetes (T2D).

**Methods:**

This study was part of a series exploring the potential risks for DPN. All patients were questioned for neurologic symptoms, examined for neurologic signs, and received nerve conduction studies to collect nerve action potential onset latency, amplitude, and nerve conduction velocity (NCV). Composite *Z* scores of latency, amplitude, and NCV were calculated. DPN was confirmed as both at least a neurologic symptom/sign and an abnormality of nerve conduction studies. Coagulation function indices, such as plasma D-dimer levels, were also synchronously detected.

**Results:**

We finally recruited 393 eligible patients for this study, of whom 24.7% (*n* = 97) were determined to have DPN. The plasma D-dimer level was found to be closely associated with the composite *Z* score of latency, amplitude, and NCV after adjusting for other coagulation function indices and clinical covariates (latency: *β* = 0.134, *t* = 2.299, *p* = 0.022; amplitude: *β* = –0.138, *t* = –2.286, *p* = 0.023; NCV: *β* = –0.139, *t* = –2.433, *p* = 0.016). Moreover, the prevalence of DPN in the first, second, third, and fourth quartiles (Q1, Q2, Q3, and Q4) of the D-dimer level was 15.2%, 15.9%, 26.4%, and 42.7%, respectively (*p* for trend < 0.001). The corresponding adjusted odds ratios and 95% CIs for DPN in D-dimer quartiles were 1, 0.79 (0.21–2.99), 1.75 (0.49–6.26), and 5.17 (1.38–19.42), respectively. Furthermore, the optimal cutoff value of the plasma D-dimer level to discriminate DPN was ≥0.22 mg/L (sensitivity = 67.01%, specificity = 58.78%, and Youden index = 0.26) after analysis by the receiver operating characteristic curve.

**Conclusions:**

Increased plasma D-dimer levels may be a promising indicator for DPN in patients with T2D.

## Introduction

Diabetic peripheral neuropathy (DPN), a primary complication of diabetes, is the main facilitative factor for falls, fractures, foot ulcerations, and amputation (1, 2). Moreover, rather than a purely peripheral neuropathy, DPN is also associated with central nervous system structural alterations (such as reduction in cervical cord cross-sectional area and somatosensory cortex gray matter volume) (3, 4) and functional abnormalities (such as abnormal thalamocortical connectivity) (5). Hence, patients with DPN are more susceptible to suffer from disability, encounter a poor quality of life, and experience reduced psychosocial wellbeing (6, 7). The pathogenesis of DPN is not very clear, but it involves the interaction between multiple factors. Therefore, it is worthwhile to explore additional risk factors for DPN, which may help develop approaches to prevent or ameliorate DPN.

D-dimer is a degradation product from fibrinolysis, and its increment in plasma serves as a traditional biomarker of hypercoagulability (8). Plasma D-dimer levels were reported to be associated with an increased risk of coronary events (8), ischemic and hemorrhagic stroke (9, 10), cardiovascular disease (CVD)-specific mortality, and cancer incidence and prognosis in the general population (11), let alone be indicative of venous thrombus formation (12). Moreover, increased plasma D-dimer levels were also documented to be partly responsible for angiopathic complications in patients with diabetes, such as microalbuminuria (13), renal dysfunction (14), diabetic retinopathy (15), atherosclerotic plaque (16), and poor cardiovascular outcomes (17). Furthermore, plasma D-dimer can be used to reflect the progressive nature of diabetes and cardiovascular complications (18). However, the relationship between plasma D-dimer and DPN in type 2 diabetes (T2D) has not been well studied. In light of the above, we hypothesized that increased plasma D-dimer levels may be a potential risk factor for DPN in T2D.

Therefore, we designed the present study to determine whether increased plasma D-dimer levels are connected to DPN in T2D.

## Methods

### Participant recruitment

This study was part of a series we designed to explore the potential risks for DPN. We recruited participants for the study from First People’s Hospital of Nantong City and Second People’s Hospital of Nantong City between November 2017 and December 2021. The inclusion criteria for participants were as follows: (1) between 20 and 80 years of age; (2) met the diagnostic criteria of T2D (2015 Edition, American Diabetes Association) (19); (3) normal coagulation function; (4) plasma D-dimer level < 2.0 mg/L; and (5) voluntarily agreed to take part in the study. The exclusion criteria for participants were described below: (1) positive for insulin antibody; (2) thyroid hormonal abnormality; (3) history of cancer; (4) severe cardio-cerebral vascular diseases (e.g., myocardial infarction); (5) chronic kidney disease, and estimated glomerular filtration rate (eGFR) < 60 ml/min/1.73 m^2^; (6) acute or chronic infectious diseases; (7) autoimmune diseases; (8) connective tissue diseases; (9) use of drugs with side effects of neurotoxicity; (10) deficiencies of folate or vitamin B12; (11) spinal or foraminal stenosis; (12) neurodegenerative diseases; (13) inflammatory demyelinating neuropathies; (14) suffered from trauma in the last 3 months; (15) history of vascular interventional surgery; (16) use of anticoagulants or antiplatelet drugs in the last 3 months; (17) thrombotic diseases; and (18) coagulopathy and platelet dysfunction. We finally recruited 393 eligible patients for this study. The study was initiated and academically supported by First People’s Hospital of Nantong, so the study protocol was reviewed and approved by the First People’s Hospital of Nantong. In addition, the processes of the study followed the Declaration of Helsinki, and all participants provided informed consent when recruited into the study.

### Clinical data collection

Clinical data from all participants were collected by trained clinical staff. These data included age, sex, body mass index (BMI), systolic/diastolic blood pressure (SBP/DBP), diabetes duration, hypertension status, statin use, and antidiabetic treatments. Hypertension was identified as reported in our previous studies (20, 21). Antidiabetic treatments in our study were divided into nine categories: drug naïve, insulin, secretagogues, metformin, thiozolindiones (TZDs), α-glucosidase inhibitors (AGIs), dipeptidyl peptidase-4 inhibitors (DPP-4Is), sodium-glucose cotransporter-2 inhibitors (SGLT-2Is), and glucagon-like peptide-1 receptor agonists (GlP-1RAs).

Plasma was isolated from blood specimens (stored by tubes with 3.2% sodium citrate solution) to detect coagulation function indices, such as international normalized ratio (INR), prothrombin time (PT), activated partial prothrombin time (APTT), fibrinogen, and D-dimer. PT, APTT, and fibrinogen were measured by the solidification method, and D-dimer was measured by the immunoturbidimetric method in a fully automated blood coagulation analyzer (CS5100, Sysmex, Japan). Serum was isolated from fasting blood samples to detect alanine aminotransferase (ALT), triglycerides (TG), total cholesterol (TC), high-density lipoprotein cholesterol (HDLC), low-density lipoprotein cholesterol (LDLC), uric acid (UA), and C-peptide. Whole blood specimens were drawn to detect glycosylated hemoglobin (HbA1c). Plasma was also isolated to detect glucagon. Serum creatinine was also detected to calculate the estimated glomerular filtration rate (eGFR) using the Modification of Diet in Renal Disease equation (22).

### Screening for DPN and nerve conduction studies

Screening for DPN was carried out as reported in our previous studies (20, 21, 23). Confirmation of DPN is dependent on both at least a neurologic symptom/sign and an abnormality of peripheral nerve conduction studies (24).

Neurological symptoms and signs were collected by detailed history taking and physical examinations. Neurologic symptoms included numbness, pain (such as tingling, stabbing, burning, shooting, and electrical shock pain), and paresthesia (such as abnormal cold or heat sensation, allodynia, and hyperalgesia), and neurologic signs were defined as reduced ankle reflexes or reduced distal sensation (such as touch sensation, thermal discrimination, nociception, vibration perception, equilibrioception, and proprioception).

Nerve conduction studies were implemented by an experienced neurological technician using an electromyogram (MEB-9200K, Nihon Kohden). The nerve conduction parameters included the onset latency, nerve action potential amplitude, and nerve conduction velocity (NCV). Motor nerve studies were conducted on two sides of the median nerve (MN), ulnar nerve (UN), common peroneal nerve (CPN), and posterior tibial nerve (PTN). Sensory nerve studies were conducted on the sides of the MN, UN, sural nerve (SN), and superficial peroneal nerve (SPN). Data of nerve latency, amplitude, and NCV were then *Z* score transformed. Furthermore, the composite *Z* score of latency was calculated by taking the average value of the latency *Z* score of all motor and sensory nerves of the upper and lower limbs, which was also described in our previous study (23). In the same way, the composite *Z* scores of amplitude and NCV were calculated.

### Statistical analysis

We used SPSS for Windows (Version 25.0, IBM Corp.) to pool and analyze clinical data, and statistical significance was identified when the *p*-value < 0.05.

First, descriptive statistics were performed for all patients and four subgroups categorized by the quartiles of plasma D-dimer levels. Means and standard deviations were for normally distributed quantitative data, medians and interquartile ranges were for skew-distributed quantitative data, and frequencies and percentages were for qualitative data. To analyze the changes in trends of clinical data among the four subgroups, one-way analysis of variance (ANOVA) with linear polynomial contrasts (*F* value), the Jonckheere–Terpstra test (standard *Z* value), and the chi-squared test with linear-by-linear association (*χ* ^2^ value) were used as appropriate. The plasma D-dimer level was skew-distributed and was natural-logarithm transformed (lnD-dimer) for further correlation and regression analysis.

Second, Pearson’s correlation was used to assess univariate correlation between plasma D-dimer and nerve conduction indices. Moreover, given that HbA1c or fibrinogen may exert an effect on these correlations, partial correlation was used to adjust the effect of HbA1c or fibrinogen on these correlations.

Third, to determine the independent effects of plasma D-dimer on nerve conduction indices, we used multivariable linear regression analyses to control for other coagulation function indices and clinical covariates. Meanwhile, we used multivariable logistic regression analyses to determine unadjusted and adjusted odds ratios (ORs) and 95% confidence intervals (CIs) for DPN in four subgroups of plasma D-dimer quartiles.

Finally, we used receiver operating characteristic (ROC) curves to assess the diagnostic capability of plasma D-dimer to confirm DPN and to determine the optimal cutoff value of plasma D-dimer to discriminate DPN. HbA1c and plasma fibrinogen were well-established risk factors for DPN in our previous studies and other previous studies (21, 25, 26). To explore whether the predictive ability of plasma D-dimer is beyond traditional risk factors, we set a reference model including age, sex, diabetes duration, BMI, SBP, DBP, hypertension, statin treatment, ALT, lipid profiles, UA, eGFR, fasting C-peptide, fasting glucagon, PT, APTT, and antidiabetic treatments. Then, we used ROC analysis to compare the performance of the reference model adding D-dimer with the reference model alone, the reference model adding fibrinogen, and the reference model adding HbA1c (methods by DeLong et al.).

## Results

### Clinical features of recruited patients

The clinical features of all eligible patients are exhibited in **Table 1**. The range of plasma D-dimer levels of all patients was 0.10–1.65 mg/L, and those of the first, second, third, and fourth quartiles (Q1, Q2, Q3, and Q4) were 0.10–0.14 mg/L, 0.15–0.22 mg/L, 0.23–0.45 mg/L, and 0.46–1.65 mg/L, respectively. In addition, with quartiles of ascending plasma D-dimer, age, SBP, diabetes duration, plasma fibrinogen, and PT were increased, while ALT, TC, LDLC, fasting C-peptide, and glucagon were decreased. However, sex distribution, DBP, hypertension history, statin use, TG, HDLC, UA, eGFR, HbA1c, and plasma APTT displayed no differences among the four subgroups. Regarding antidiabetic treatments, insulin use tended to increase plasma D-dimer within a certain range (*p* = 0.041), while SGLT-2Is use tended to decrease plasma D-dimer, but this failed to reach statistical significance (*p* = 0.052). However, drug naïve, uses of secretagogues, metformin, TZDs, AGIs, DPP-4Is, and GLP-1RAs were comparable among the four subgroups.

**Table 1:** Clinical features of the recruited patients.

Variables	Total	Quartiles of plasma D-dimer levels				Test statistic	*p* for trend
		Q1	Q2	Q3	Q4		
Plasma D-dimer (mg/L) (range)	0.36 ± 0.32 (0.10–1.65)	0.11 ± 0.02 (0.10–0.14)	0.19 ± 0.02 (0.15–0.22)	0.32 ± 0.07 (0.23–0.45)	0.86 ± 0.28 (0.46–1.65)	–	–
ln D-dimer	–1.33 ± 0.76	–2.18 ± 0.13	–1.69 ± 0.11	–1.16 ± 0.20	–0.20 ± 0.32	–	–
*n*	393	99	107	91	96	–	–
Age (years)	51.4 ± 8.9	49.3 ± 6.4	50.5 ± 8.9	53.2 ± 9.1	53.0 ± 10.4	11.767**^a^**	0.001
Female, *n* (%)	157 (39.9)	39 (39.4)	40 (37.4)	33 (36.3)	45 (46.9)	0.938**^c^**	0.333
BMI (kg/m^2^)	25.1 ± 3.2	25.3 ± 3.0	25.4 ± 3.0	25.2 ± 3.4	24.5 ± 3.3	3.152**^a^**	0.077
SBP (mmHg)	132.8 ± 16.3	129.9 ± 17.8	133.0 ± 15.4	133.2 ± 16.5	135.2 ± 15.4	4.849**^a^**	0.028
DBP (mmHg)	79.7 ± 10.5	78.8 ± 9.9	81.7 ± 11.4	79.8 ± 9.1	78.1 ± 11.0	0.714**^a^**	0.398
Diabetes duration (years)	5.0 (1.0–10.0)	5.0 (1.0–9.5)	4.0 (1.0–8.3)	8.0 (1.0–12.0)	7.5 (1.3–10.0)	2.486**^b^**	0.013
Antidiabetic treatments							
Drug naive, *n* (%)	41 (10.4)	11 (11.1)	13 (12.1)	9 (9.9)	8 (8.3)	0.591**^c^**	0.442
Insulin, *n* (%)	166 (42.2)	36 (3.4)	43 (40.2)	38 (41.8)	49 (51.0)	4.160**^c^**	0.041
Secretagogues, *n* (%)	172 (43.8)	45 (45.5)	37 (34.6)	46 (50.5)	44 (45.8)	0.596**^c^**	0.440
Metformin, *n* (%)	192 (48.9)	52 (52.5)	53 (49.5)	48 (52.7)	39 (40.6)	2.067**^c^**	0.151
TZDs, *n* (%)	73 (18.6)	19 (19.2)	16 (15.0)	18 (19.9)	20 (20.8)	0.314**^c^**	0.571
AGIs, *n* (%)	54 (13.7)	12 (12.1)	15 (14.0)	10 (11.0)	17 (17.7)	0.778**^c^**	0.378
DPP-4Is, *n* (%)	60 (15.3)	18 (18.2)	19 (17.8)	12 (13.2)	11 (11.5)	2.311**^c^**	0.128
SGLT-2Is, *n* (%)	16 (4.1)	7 (7.1)	5 (4.7)	2 (2.2)	2 (2.1)	3.774**^c^**	0.052
GLP-1RAs, *n* (%)	31 (7.9)	6 (6.1)	8 (7.5)	6 (6.6)	11 (11.5)	1.577**^c^**	0.209
Hypertension, *n* (%)	143 (36.4)	33 (33.3)	36 (33.6)	40 (44.0)	34 (35.4)	0.572**^c^**	0.449
Statins uses, *n* (%)	117 (29.8)	28 (28.3)	23 (21.5)	34 (37.4)	32 (33.3)	2.266**^c^**	0.132
ALT (U/L)	19 (13–28)	22 (13–28)	21 (14–31)	13 (20–29)	15 (11–22)	–2.738**^b^**	0.006
TG (mmol/L)	1.64 (1.04–2.51)	1.73 (1.19–2.44)	1.72 (1.14–2.78)	1.61 (0.86–2.87)	1.45 (1.05–2.34)	–1.421**^b^**	0.155
TC (mmol/L)	4.38 ± 0.96	4.52 ± 1.06	4.54 ± 0.85	4.17 ± 0.92	4.26 ± 0.98	6.575**^a^**	0.011
HDLC (mmol/L)	1.17 ± 0.36	1.17 ± 0.28	1.18 ± 0.53	1.15 ± 0.29	1.18 ± 0.26	0.008**^a^**	0.927
LDLC (mmol/L)	2.73 ± 0.85	2.87 ± 0.95	2.89 ± 0.74	2.50 ± 0.77	2.63 ± 0.87	7.840**^a^**	0.005
UA (μmol/L)	298 ± 88	303 ± 101	299 ± 82	296 ± 79	294 ± 92	0.423**^a^**	0.516
Fasting C-peptide (ng/ml)	1.44 (0.86–2.19)	1.73 (0.97–2.21)	1.62 (0.86–2.41)	1.37 (0.85–2.05)	1.14 (0.69–1.73)	–3.042**^b^**	0.002
Fasting glucagon (pg/ml)	148.1 (113.3–202.0)	161.0 (115.1–208.6)	161.0 (119.1–222.5)	134.9 (111.8–210.2)	136.2 (111.8–177.5)	–2.180**^b^**	0.029
eGFR (ml/min/1.73 m^2^)	120 ± 34	119 ± 29	125 ± 32	122 ± 44	114 ± 30	1.124**^a^**	0.290
HbA1c (%)	8.09 ± 1.17	7.87 ± 1.10	8.12 ± 1.20	8.22 ± 1.26	8.15 ± 1.10	3.243**^a^**	0.073
Plasma fibrinogen (g/L)	2.50 ± 0.78	2.22 ± 0.57	2.47 ± 0.56	2.54 ± 0.68	2.78 ± 0.85	33.037**^a^**	<0.001
Plasma PT (s)	11.36 ± 0.88	11.32 ± 1.00	11.10 ± 0.75	11.41 ± 0.89	11.65 ± 0.81	11.325**^a^**	0.001
Plasma APTT (s)	29.46 ± 5.16	29.97 ± 5.58	28.73 ± 4.38	29.83 ± 5.71	29.41 ± 4.95	0.057**^a^**	0.811
Composite *Z* score of latency	0.03 ± 0.61	–0.08 ± 0.57	–0.11 ± 0.55	0.06 ± 0.59	0.27 ± 0.68	20.255**^a^**	<0.001
Composite *Z* score of amplitude	–0.02 ± 0.67	0.19 ± 0.64	0.02 ± 0.58	–0.11 ± 0.70	–0.21 ± 0.67	20.598**^a^**	<0.001
Composite *Z* score of NCV	–0.03 ± 0.74	0.19 ± 0.67	0.13 ± 0.58	–0.11 ± 0.85	–0.34 ± 0.77	32.079**^a^**	<0.001
DPN, *n* (%)	97 (24.7)	15 (15.2)	17 (15.9)	24 (26.4)	41 (42.7)	22.855**^c^**	<0.001

**^a^** Linear polynomial contrasts of ANOVA (F value), **^b^** Jonckheere–Terpstra test (Z value), and **^c^** linear-by-linear association of chi-squared test (χ^2^ value) were performed as appropriate.

### Correlations between plasma D-dimer and nerve conduction indices

**Figure 1:**
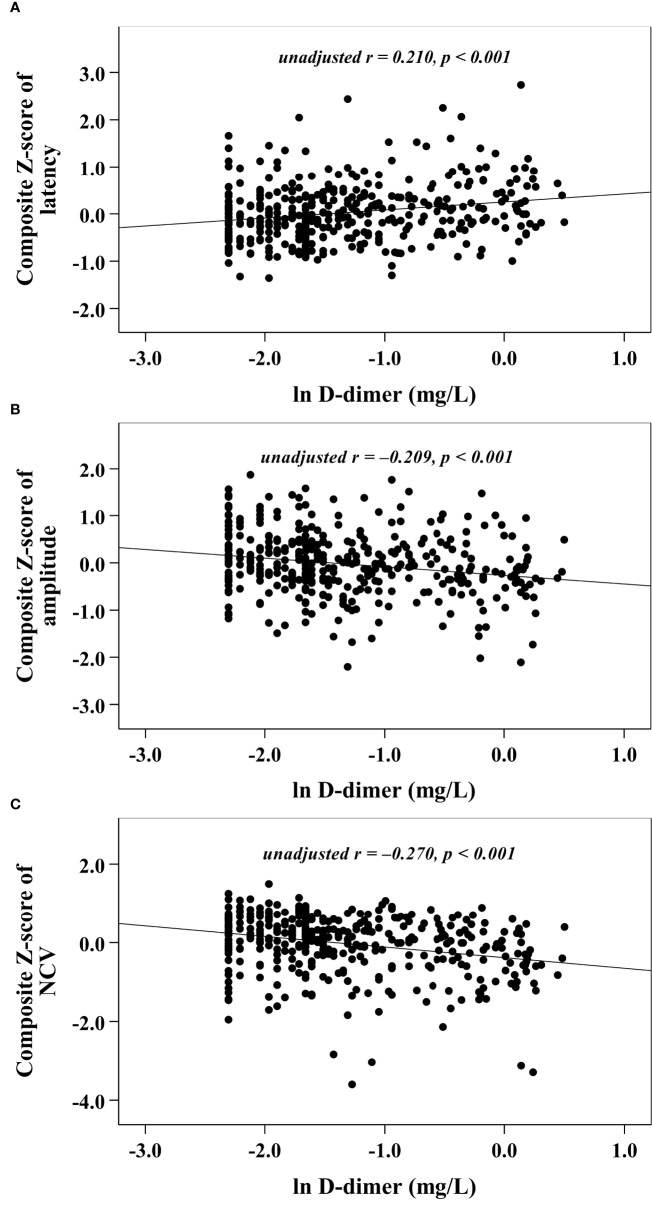
Graphically exhibited correlations between D-dimer and nerve conduction indices (**A:** composite Z score of latency; **B:** composite Z score of amplitude; **C:** composite Z score of NCV).

**Figure 2:**
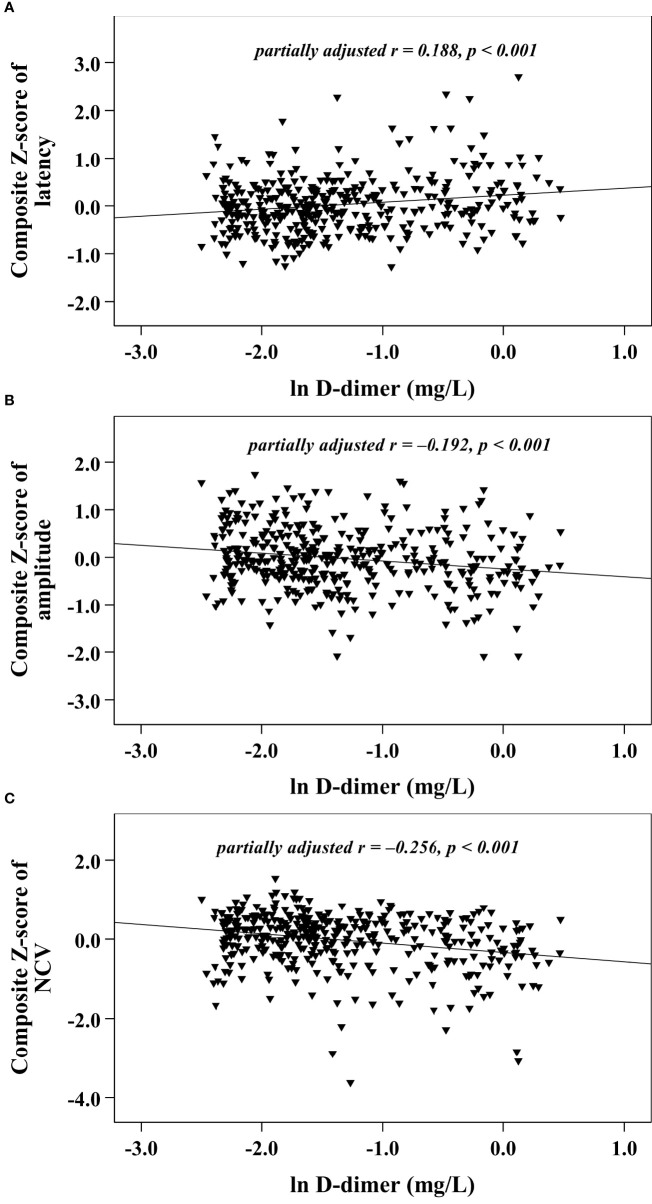
Graphically exhibited correlations between D-dimer and nerve conduction indices after adjusting for HbA1c (**A:** composite Z score of latency; **B:** composite Z score of amplitude; **C:** composite Z score of NCV).

**Figure 3:**
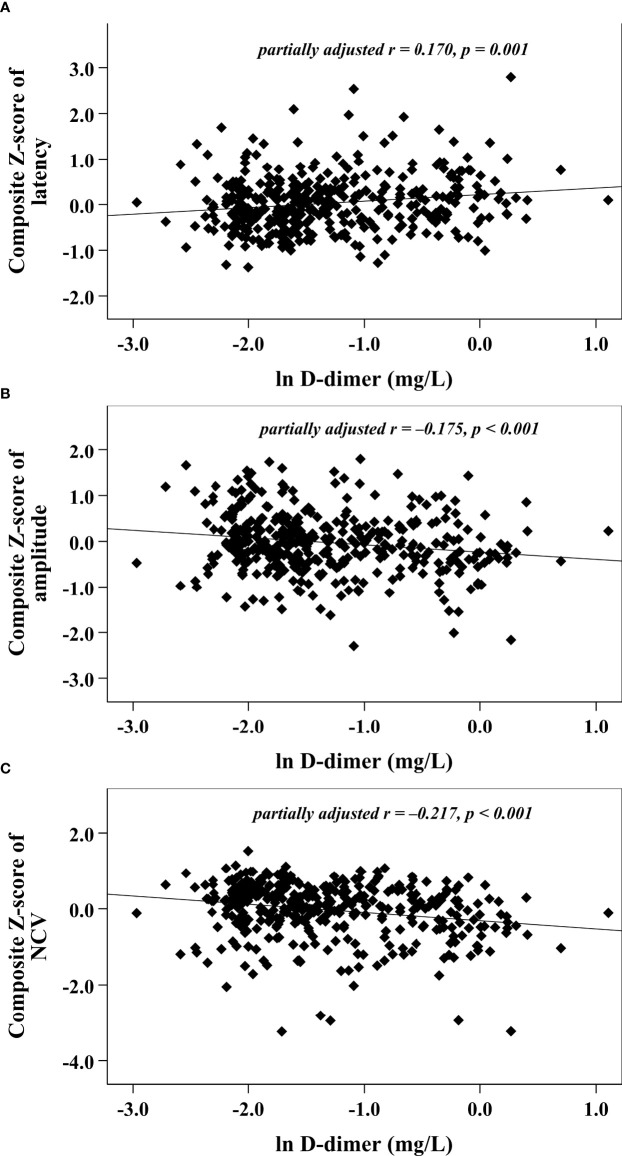
Graphically exhibited correlations between D-dimer and nerve conduction indices after adjusting for fibrinogen (**A:** composite Z score of latency; **B:** composite Z score of amplitude; **C:** composite Z score of NCV).

With increasing quartiles of plasma D-dimer, the composite *Z* score of latency was increased, while the composite *Z* score of amplitude and NCV were markedly decreased (**Table 1**). Univariate correlation analysis demonstrated that the plasma D-dimer level was linked to the composite *Z* score of nerve latency, amplitude and NCV (*r* = 0.210, –0.209, and –0.270, respectively, *p* < 0.001) (**Figure 1**). After controlling for the potential effect of HbA1c on these correlations by partial correlation analysis, plasma D-dimer levels remained linked to composite *Z* scores of nerve latency, amplitude, and NCV (*r* = 0.188, –0.192, and –0.256, respectively, *p* < 0.001) (**Figure 2**). Meanwhile, after controlling for the potential effect of fibrinogen on these correlations, plasma D-dimer levels remained linked to composite *Z* scores of nerve latency, amplitude and NCV (*r* = 0.170, –0.175, and –0.217, respectively, *p* < 0.001) (**Figure 3**).

Moreover, we used multivariable linear regression analyses to determine the effects of plasma D-dimer on nerve conduction indices (**Table 2**). After gradually adjusting for other coagulation function indices and clinical covariates (from model 0 to model 4), plasma D-dimer levels remained independently associated with nerve conduction indices. The fully adjusted model 4 demonstrated that plasma D-dimer level was independently and positively related to the composite *Z* score of latency (*β* = 0.134, *t* = 2.299, *p* = 0.022) and was independently and negatively related to the composite *Z* score of amplitude (*β* = –0.138, *t* = –2.286, *p* = 0.023) and NCV (*β* = –0.139, *t* = –2.433, *p* = 0.016), respectively.

**Table 2:** Impacts of plasma D-dimer on outcomes of nerve conduction indices by multivariable linear regression analysis.

Models	B (95% CI)	*β*	*t*	*p*	Adjusted *R* ^2^
**Composite *Z* score of latency**					
Model 0	0.169 (0.091 to 0.248)	0.210	4.234	<0.001	0.044
Model 1	0.150 (0.077 to 0.223)	0.185	4.022	<0.001	0.236
Model 2	0.149 (0.065 to 0.243)	0.183	3.478	0.001	0.400
Model 3	0.151 (0.065 to 0.237)	0.184	3.452	0.001	0.432
Model 4	0.109 (0.016 to 0.203)	0.134	2.299	0.022	0.443
**Composite *Z* score of amplitude**					
Model 0	–0.182 (–0.267 to –0.097)	–0.209	–4.129	<0.001	0.044
Model 1	–0138 (–0.217 to –0.059)	–0.158	–3.440	0.001	0.237
Model 2	–0.147 (–0.244 to –0.049)	–0.160	–2.970	0.003	0.368
Model 3	–0.134 (–0.233 to –0.035)	–0.147	–2.677	0.008	0.402
Model 4	–0.127 (–0.235 to –0.018)	–0.138	–2.286	0.023	0.405
**Composite *Z* score of NCV**					
Model 0	–0.269 (–0.363 to –0.175)	–0.274	–5.639	<0.001	0.075
Model 1	–0.234 (–0.325 to –0.143)	–0.239	–5.040	<0.001	0.191
Model 2	–0.209 (–0.316 to –0.103)	–0.206	–3.888	<0.001	0.389
Model 3	–0.186 (–0.293 to –0.079)	–0.183	–3.429	0.001	0.430
Model 4	–0.142 (–0.256 to –0.027)	–0.139	–2.433	0.016	0.461

D-dimer was natural logarithmically transformed for the regression analysis.Model 0: unadjusted.

Model 1: adjusted for age, sex, diabetic duration, BMI, SBP, DBP, hypertension and statins treatment.

Model 2: additionally adjusted for ALT, lipid profiles, UA, eGFR, HbA1c, fasting C-peptide and glucagon.

Model 3: additionally adjusted for antidiabetic treatments.

Model 4: additionally adjusted for plasma fibrinogen, PT and APTT.

### Risks for DPN at differential levels of plasma D-dimer quartiles

After DPN assessment, 24.7% (*n* = 97) of recruited patients were determined to have DPN. The prevalence of DPN in Q1, Q2, Q3, and Q4 of the D-dimer level was 15.2%, 15.9%, 26.4%, and 42.7%, respectively (*p* for trend < 0.001). Moreover, the ORs and 95% CIs for DPN in Q1, Q2, Q3, and Q4 of the D-dimer level were 1, 1.06 (0.50–2.25), 2.01 (0.98–4.12), and 4.18 (2.11–8.26), respectively (**Table 3**). Furthermore, after adjusting for other coagulation function indices and clinical covariates by multivariable logistic regression analyses, the corresponding ORs and 95% CIs for DPN in the Q1, Q2, Q3, and Q4 plasma D-dimer quartiles were 1, 0.79 (0.21–2.99), 1.75 (0.49–6.26), and 5.17 (1.38–19.42), respectively (**Table 3**).

**Table 3:** Risks for DPN at differential levels of plasma D-dimer quartiles (ORs [95% CIs]).

Models	Q1	Q2	Q3	Q4	*p* value for trend
*n*	99	107	91	96	–
DPN, *n* (%)	15 (15.2)	17 (15.9)	24 (26.4)	41 (42.7)	–
Model 0	1–reference	1.06 (0.50 to 2.25)	2.01 (0.98 to 4.12)	4.18 (2.11 to 8.26)	<0.001
Model 1	1–reference	1.04 (0.48 to 2.25)	1.65 (0.78 to 3.50)	3.78 (1.86 to 7.70)	<0.001
Model 2	1–reference	1.14 (0.34 to 3.79)	1.60 (0.50 to 5.18)	6.10 (1.98 to 18.84)	0.001
Model 3	1–reference	1.06 (0.30 to 3.78)	2.15 (0.62 to 7.43)	7.05 (2.10 to 23.63)	<0.001
Model 4	1–reference	0.79 (0.21 to 2.99)	1.75 (0.49 to 6.26)	5.17 (1.38 to 19.42)	0.005

Model 0: unadjusted.

Model 1: adjusted for age, sex, diabetes duration, BMI, SBP, DBP, hypertension, and statin treatment.

Model 2: additionally adjusted for ALT, lipid profiles, UA, eGFR, HbA1c, fasting C-peptide, and glucagon.

Model 3: additionally adjusted for antidiabetic treatments.

Model 4: additionally adjusted for plasma fibrinogen, PT, and APTT.

### Potential capability of plasma D-dimer to discriminate DPN

**Figure 4:**
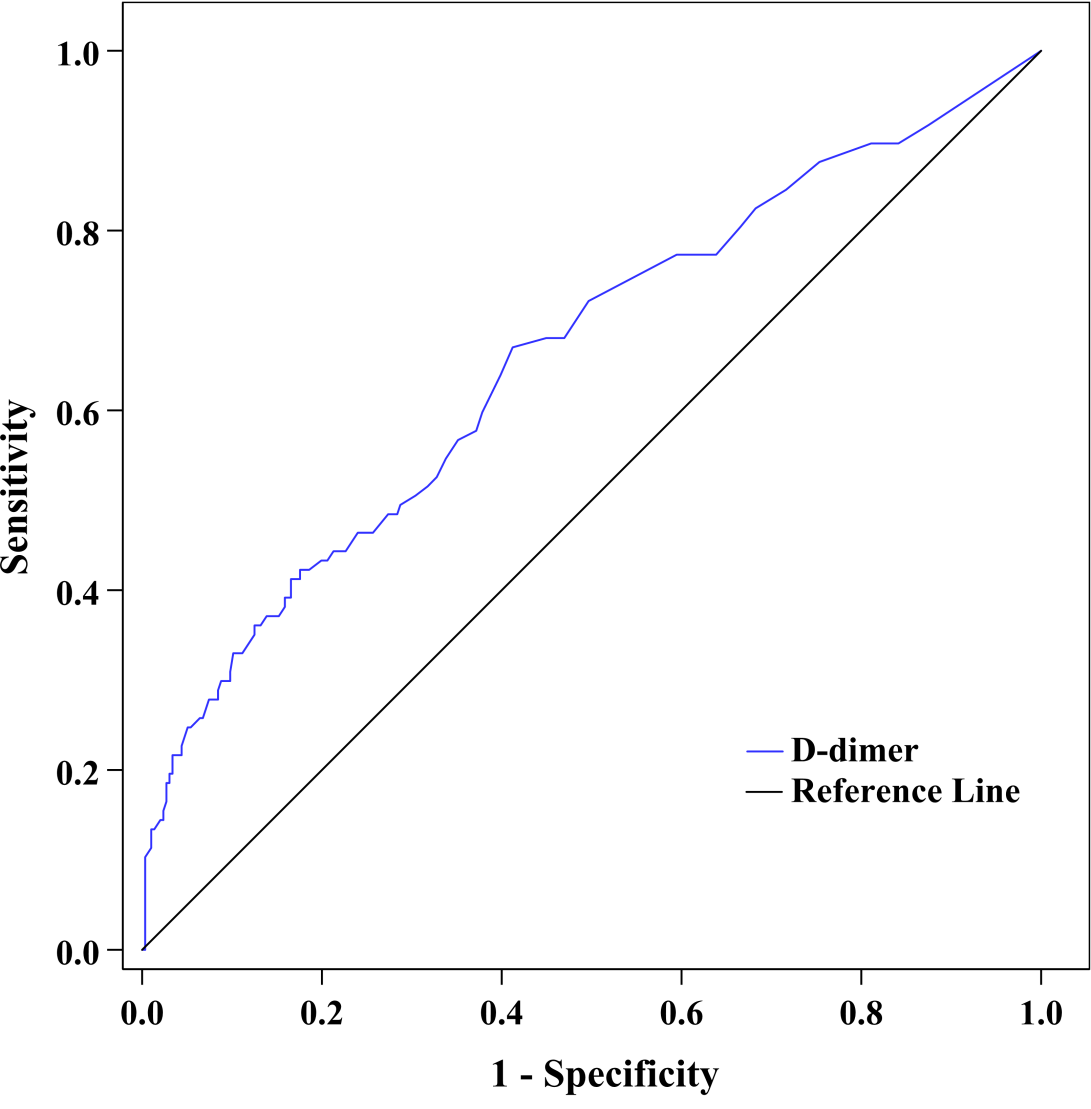
ROC curve exhibited the capability of plasma D-dimer to discriminate DPN (AUC was 0.659 [95% CI: 0.610–0.706], optimal cutoff value was ≥0.22 mg/L, Youden index was 0.26, sensitivity was 67.01%, and specificity was 58.78%).

**Figure 4** exhibits the capability of plasma D-dimer to discriminate DPN after ROC curve analysis. The area under the ROC curve (AUC) of plasma D-dimer was 0.659 (95% CI: 0.610–0.706). Additionally, ROC analysis also determined that the optimal cutoff value of plasma D-dimer to discriminate DPN was ≥0.22 mg/L, with a Youden index of 0.26, a sensitivity of 67.01%, and a specificity of 58.78%.

### Performance of plasma D-dimer to discriminate DPN after adjustment for traditional risk factors

**Figure 5:**
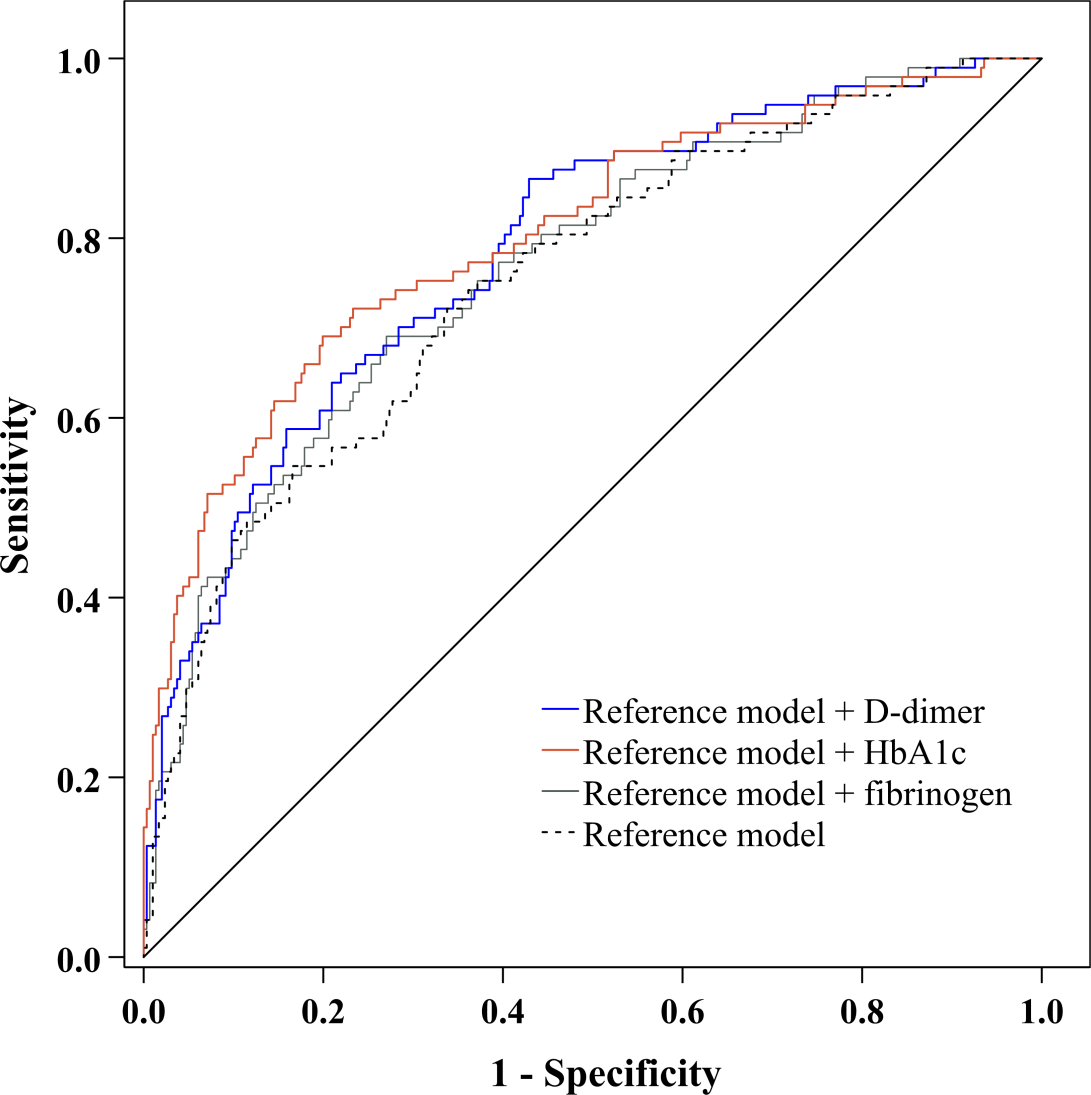
ROC curve exhibited the performance of plasma D-dimer to discriminate DPN after adjustment for traditional risk factors.

Moreover, to explore whether the predictive ability of plasma D-dimer to discriminate DPN is beyond traditional risk factors, we used ROC analysis to compare the performance of the reference model adding D-dimer with the reference model alone, the reference model adding fibrinogen, and the reference model adding HbA1c (**Figure 5**). After comparing the AUC for these models, we found that the performance of the reference model adding D-dimer to discriminate DPN was superior to that of the reference model alone (**Table 4**). However, the performance of the reference model adding D-dimer, the reference model adding HbA1c, and the reference model adding fibrinogen was comparable to discriminate DPN (**Table 4**).

**Table 4:** Performance of plasma D-dimer to discriminate DPN after adjustment for traditional risk factors.

Models	AUC (95% CI)	AUC differences (95% CI)	*Z* for AUC differences	*p* for AUC differences
Reference model + D-dimer	0.786 (0.742 to 0.825)	–		
Reference model	0.754 (0.708 to 0.795)	0.0324 (0.0008 to 0.0640)**^a^**	2.010	0.041
Reference model + fibrinogen	0.767 (0.722 to 0.808)	0.0193 (–0.0095 to 0.0482)**^b^**	1.313	0.189
Reference model + HbA1c	0.802 (0.759 to 0.841)	0.0164 (–0.0316 to 0.0644)**^c^**	0.670	0.503

The reference model includes age, sex, diabetes duration, BMI, SBP, DBP, hypertension, statin treatment, ALT, lipid profiles, UA, eGFR, fasting C-peptide, fasting glucagon, PT, APTT, and antidiabetic treatments.

**^a^** Reference model + D-dimer vs. reference model.

**^b^** Reference model + D-dimer vs. reference model + fibrinogen.

**^c^** Reference model + D-dimer vs. reference model + HbA1c.

## Discussion

DPN is extremely complex and debilitating due to its pathogenesis involving a complex interaction between multiple factors. We initiated a series to find additional risk factors for DPN. The present study aimed to determine whether plasma D-dimer is an independent risk factor for DPN in T2D. The primary contributions of the study are described as follows: first, plasma D-dimer was closely connected with the nerve action potential onset latency, amplitude, and conduction velocity, even after correcting for clinical covariates; second, the risk of DPN was estimated to be fivefold (OR 5.17, 95% CI: 1.38–19.42) higher in patients in the highest quartile of plasma D-dimer than in those in the lowest quartile; third, plasma D-dimer ≥0.22 mg/L was the optimal cutoff value to discriminate DPN (sensitivity = 67.01%, specificity = 58.78%); fourth, compared to the well-established risk factors (reference model adding HbA1c and reference model adding plasma fibrinogen), we found that plasma D-dimer, HbA1c, and fibrinogen were comparable in the ability to discriminate DPN.

DPN incidence and progression result from the accumulation of a complex interaction of multiple cardiometabolic risk factors in the background of diabetes (27, 28). Previous well-conducted studies have shown that glucose burden is a central risk factor underlying the pathogenesis of diabetic neuropathy (25). Other risk factors have also been identified to play an important role in diabetic neuropathy, such as long-term diabetes, aging, abdominal obesity, hypertension, smoking, dyslipidemia (raised TG level and decreased HDLC level), hypoalbuminemia, and anemia (2, 29–32). Of course, glucose burden is the most notable risk factor for DPN, and our previous series demonstrated that short-term glycemic fluctuation estimated by the mean amplitude of glycemic excursions (MAGE) (21) and plasma 1,5-anhydro-d-glucitol (23) and long-term glycemic fluctuation estimated by HbA1c variability (20) were involved in DPN. Alongside an excessive glucose burden, a hypercoagulability state was also found to take part in the vascular complications of diabetes (33). As a coagulation factor, an increased level of plasma fibrinogen was revealed to be closely related to DPN (26). In our present study, plasma D-dimer, a degradation product of fibrinogen, was found to be associated with DPN. We also found that plasma D-dimer, HbA1c, and fibrinogen were comparable in their ability to indicate DPN.

Increased plasma D-dimer levels not only can predict a range of adverse health outcomes in the general population (11, 34) but also can partly account for metabolic diseases and vascular complications with a background of insulin resistance. When compared to the healthy controls, the level of plasma D-dimer was obviously elevated in the first-degree relatives of T2D (35), prediabetes (18), gestational hypertension (36), polycystic ovary syndrome (37), and metabolic syndrome (38), let alone in overt T2D. Moreover, plasma D-dimer was observed to be independently associated with inflammatory cytokines (39), oxidized LDL (40), poor glycemic control (hyperglycemia, glycemic variability, and hypoglycemia) (41–43), diabetic retinopathy and nephropathy (14, 15), and CVD (17) in patients with T2D. In addition, plasma D-dimer levels were also increased in parallel with the differential stages from family history of diabetes to prediabetes to T2D with CVD complications (18), which suggested that plasma D-dimer could be applied to indicate the progressive nature of diabetes and diabetes-related complications. Furthermore, in our present study, increased plasma D-dimer levels were revealed to be associated with increased nerve action potential onset latency and decreased nerve amplitude and NCV in T2D. In addition, patients in the highest quartile of plasma D-dimer presented with a fivefold higher DPN risk than those in the lowest quartile. We also calculated that plasma D-dimer ≥0.22 mg/L was the optimal cutoff value to discriminate DPN, with a sensitivity of 67.01% and a specificity of 58.78%. Our study added evidence to support increased plasma D-dimer in the pathogenesis of diabetic microvascular complications.

Several underlying mechanisms may explain the correlation of increased plasma D-dimer with DPN in T2D. In the context of T2D, hyperglycemic exposure and dyslipidemia together with microvascular disease trigger detrimental downstream oxidative stress (44), mitochondrial damage, and inflammation in peripheral neurons, glial cells, and vascular endothelial cells, all of which may result in impaired nerve function and neuropathy (27, 28). Increased plasma D-dimer reflects a hypercoagulability state (45), which may potentiate microvascular disease and endothelial dysfunction, leading to subsequent insufficient neural blood flow, endoneurial hypoxia, and neuronal damage (28, 33). Plasma D-dimer is also a thromboinflammatory factor (45). A previous study showed that D-dimer can stimulate monocytes *in vitro* to release inflammatory cytokines (such as IL-1β and IL-6), which indicates that D-dimer may facilitate localized coagulation (46). Moreover, increased plasma D-dimer was also found to be independently associated with an inflammatory state (assessed by IL-6) and dyslipidemia in T2D (39, 40), which could contribute to nerve dysfunction and neuropathy. Furthermore, increased plasma D-dimer was also a marker of psychosocial distress (47, 48), which has been shown to participate in the maladaptations of the peripheral and central nervous systems (49, 50).

The present study should be addressed in light of a few limitations. First, our study was cross-sectionally conducted and may not conclude a causal relationship between elevated plasma D-dimer and DPN. We need a longitudinal study to compensate for this defect. Second, we did not find a relationship between increased plasma D-dimer and the severity of DPN evaluated by the Michigan neuropathy screening instrument (MNSI). The possible causes are that the sample size is small (97 cases with DPN), and the MNSI tends to be influenced by subjective factors. Third, the duration of DPN was difficult to clearly define, so the data were not presented and analyzed in our study. Fourth, plasma D-dimer levels were determined by one sampling. However, the plasma D-dimer level could fluctuate during the course of T2D and might be affected by antidiabetic medications. It would be more convincing to present the average data of plasma D-dimer collected within weeks or months. Fifth, the level of plasma D-dimer can be affected by hypoglycemia (43), and we did not assess the effect of hypoglycemic events on the relationship between plasma D-dimer and DPN. Sixth, our results may be affected by the heterogeneities of T2D patients who received multiple antidiabetic agents. We tried our best to compensate for this limitation by adjusting for antidiabetic agents during the statistical analysis.

## Conclusion

In summary, increased plasma D-dimer levels were closely connected to dysfunction of peripheral nerve conduction and prevalence of DPN in T2D and could serve as a promising indicator for DPN in T2D.

## Data availability statement

The original contributions presented in the study are included in the article/supplementary material. Further inquiries can be directed to the corresponding author.

## Ethics statement

The studies involving human participants were reviewed and approved by First People’s Hospital of Nantong. The patients/participants provided their written informed consent to participate in this study.

## Author contributions

J-bS and X-qW initiated and acquired funding for the series; LZ, CY, and J-bS designed this study; D-mZ coordinated and supervised the study; CY, LZ, FX, L-hZ, X-hW, C-hW, L-yN, and X-lZ recruited patients and collected the data; LZ, CY, and J-bS analyzed the data and interpreted the results; LZ and CY drafted the manuscript; all authors read, modified, and approved the manuscript.

## Funding

The study was funded by Social Development Projects of Nantong (MS22015065, MS12019019, HS2020005, and JC2020045), the Medical Research Project of Nantong Health Commission (MB2020029), and the Medical Research Project of Jiangsu Health Commission (QNRC2016408).

## Conflict of interest

The authors declare that the research was conducted in the absence of any commercial or financial relationships that could be construed as a potential conflict of interest.

## Publisher’s note

All claims expressed in this article are solely those of the authors and do not necessarily represent those of their affiliated organizations, or those of the publisher, the editors and the reviewers. Any product that may be evaluated in this article, or claim that may be made by its manufacturer, is not guaranteed or endorsed by the publisher.

## Abbreviations

T2D, type 2 diabetes; DPN, diabetic peripheral neuropathy; lnD-dimer, natural logarithm transformed D-dimer; NCV, nerve conduction velocity; SBP/DBP, systolic/diastolic blood pressure; BMI, body mass index; TZDs, thiozolindiones; AGIs, α-glucosidase inhibitors; DPP-4Is, dipeptidyl peptidase-4 inhibitors; SGLT-2Is, sodium-glucose cotransporter-2 inhibitors; GLP-1RAs, glucagon-like peptide-1 receptor agonists; ALT, alanine aminotransferase; TG, triglycerides; TC, total cholesterol; HDLC, high-density lipoprotein cholesterol; LDLC, low-density lipoprotein cholesterol; UA, uric acid; eGFR, estimated glomerular filtration rate; HbA1c, glycosylated hemoglobin A1c; PT, prothrombin time; APTT, activated partial prothrombin time; MN, median nerve; UN, ulnar nerve; CPN, common peroneal nerve; PTN, posterior tibial nerve; SN, sural nerve; SPN, superficial peroneal nerve.
